# Molecular characterization of small supernumerary marker chromosomes derived from chromosome 14/22 detected in adult women with fertility problems: Three case reports and literature review

**DOI:** 10.1097/MD.0000000000022532

**Published:** 2020-10-02

**Authors:** Meiling Sun, Han Zhang, Qi Xi, Leilei Li, Xiaonan Hu, Hongguo Zhang, Ruizhi Liu

**Affiliations:** a Center for Reproductive Medicine and Center for Prenatal Diagnosis, First Hospital; b Jilin Engineering Research Center for Reproductive Medicine and Genetics, Jilin University, Changchun, Jilin, P.R. China.

**Keywords:** chromosome microarray analysis, fertility problems, fluorescence in situ hybridization, molecular cytogenetics, small supernumerary marker chromosomes (14/22)

## Abstract

**Rationale:**

Small supernumerary marker chromosomes (sSMC) are structurally abnormal chromosomes, which can be detected in patients with developmental retardation, infertile problems, and prenatal fetus. We report 3 adult female with fertility problems carrying sSMC(14/22) and aim to explore the correlation between sSMC(14/22) and fertility problems in women.

**Patient concerns:**

Three Chinese female patients were referred for infertility consultation in our hospital.

**Diagnoses:**

The karyotype of these 3 patients were 47, XX, +mar. The chromosome microarray analysis (CMA) detected various chromosomal duplications and deletions in the 3 cases: a 0.49Mb gain of 5q32 for case 1; a 0.54Mb gain of 14q32.33 and a 1.83Mb gain of 16p11.2 for case 2; a 0.37Mb loss of 13q21.2q21.31 and a 0.12Mb gain of Xp11.2 for case 3. Fluorescence in situ hybridization (FISH) using the specific probes for chromosomes 13/21, 14/22, and 15 was applied to identify the origination of these sSMC, which were all finally identified as sSMC(14/22).

**Interventions:**

Case 1 underwent the artificial reproductive technology to get her offspring and finally delivered a healthy male infant at 39 weeks. Case 2 did not plan to choose in vitro fertilization (IVF) to get offspring. Case 3 refused to do assisted reproductive technology.

**Outcomes:**

The genotype–phenotype correlation of sSMC(14/22) remain unclear. However, the existence of sSMC(14/22) might negatively affect the fertility ability in sSMC female carriers.

**Lessons:**

The combined application of traditional banding technique and molecular cytogenetic techniques can better identify more details of sSMC. For sSMC carriers with fertility problems, they could get their offsprings through assisted reproductive technologies after comprehensive fertility assessment.

## 1 Introduction

Small supernumerary marker chromosomes (sSMC) were structurally abnormal chromosomes which were too small to be identified by traditional cytogenetics only.[^1^,^2^] The detecting incidence of sSMC was 0.075% in prenatal diagnosis and there were approximately 2.7 million living sSMC carriers worldwide.^[2]^ About 70% sSMC carriers were developmentally normal and around 30% sSMC carriers manifested clinical anomalies. The sSMC frequencies were 0.288% in patients with mental retardation and 0.125% in infertile patients.[^1^,^2^,^3^] Influencing factors, such as the shapes, degree of mosaicism, origins, inheritance, and genetic contents, were associated with the diverse and complex manifestations of sSMC carriers.^[4]^

Most sSMC consisted of the short arms and pericentric region that were derived from acrocentric chromosomes.^[5]^ sSMC(15) were the most common type of in all sSMC carriers who generally presented normal phenotype.^[6]^ In prenatal cases, de novo sSMC were no way to be predicted precisely the outcome. Approximately 7% de novo sSMC deriving from chromosomes 13/21 and 14/22 resulted in an abnormal phenotype.^[7]^ Dysmorphic features and mental retardation were the common clinical features in reported sSMC(14) patients.^[8]^ As for sSMC(22), the non-mosaic dicentric duplications of the euchromatic region 22q11.2 was the most common form which was accounting for 80% in sSMC(22) carriers, and the pathologic phenotypes included cat-eye syndrome, Emanuel syndrome, or malignant tumors.[^8^,^9^]

Here, we reported 3 women non-mosaic sSMC(14/22) carriers with fertility problems and no other abnormal clinical phenotypes. Traditional cytogenetic combined with molecular genetic analysis provided a detailed analysis for these sSMC. Meanwhile, we made a review on the adult women presenting non-mosaic sSMC(14/22) with fertility problems based upon the sSMC database.

## 2 Methods

Three women were referred to the Center for Reproductive Medicine and the Center for Prenatal Diagnosis of First Hospital of Jilin University (Changchun, China) for fertility consultation. A series of examinations were performed on the 3 women and the clinical information were shown in Table 1. The study protocol was approved by the Ethics Committee of the First Hospital of Jilin University (2014–334), and the informed written consents were obtained from all the women for publication of this case report and accompanying images.

**Table 1:**
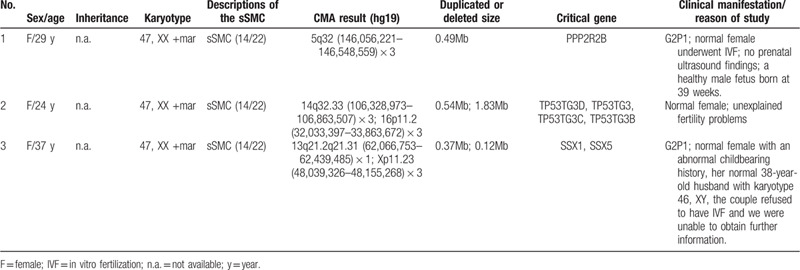
Clinical manifestation of present 3 cases.

### 2.1 Cytogenetic analysis

G-banding analysis was performed on the cultured peripheral blood cells and the cultured amniotic fluid cells at 300 to 400 banding resolution, according to standard protocols. Fifty metaphases were analyzed for all samples. We described the karyotype according to the International System for Human Cytogenetic Nomenclature (ISCN 2016).

### 2.2 Chromosomal microarray analysis (CMA)

CMA was performed in accordance with the manufacturer's protocol by CytoScan 750K array (Affymetrix, Santa Clara, CA). This method detects the human genomic DNA copy number variations (CNVs) and loss of heterozygosity (LOH) with ≥50 probe labels and ≥200Kb resolution, covering 22 pairs of autosomal and sex chromosomes. Thresholds for genome-wide screening were set at ≥200 kb for gains, ≥100 kb for losses, and ≥10 Mb for LOH. The detected CNVs were exhaustively evaluated for clinical significance through the published literature and the public database: DECIPHER, database of genomic variants (DGV), Online Mendelian Inheritance in Man (OMIM), and so on.^[10]^

### 2.3 Fluorescence in situ hybridization (FISH)

Specific probes for chromosomes 13/21, 14/22, and 15 were used to further investigate the origins of the sSMC. The D13Z1 probe was located at 13p11.1-q11.1 (spectrum green), the D21Z1 probe was located at 21p11.1-q11.1 (spectrum green), the D14Z1 probe was located at 14p11.1-q11.1 (spectrum red), the D22Z1 probe was located at 22p11.1-q11.1 (spectrum red), the D15Z1 probe was located at the 15p11.2 (spectrum green), the SNRPN probe was located at 15q11-q13 (spectrum orange), and the PML probe was located at 15q24 (spectrum orange) (Cytocell Technologies, Cambridge, UK).^[11]^

## 3 Case report

### 3.1 Case 1

**Figure 1:**
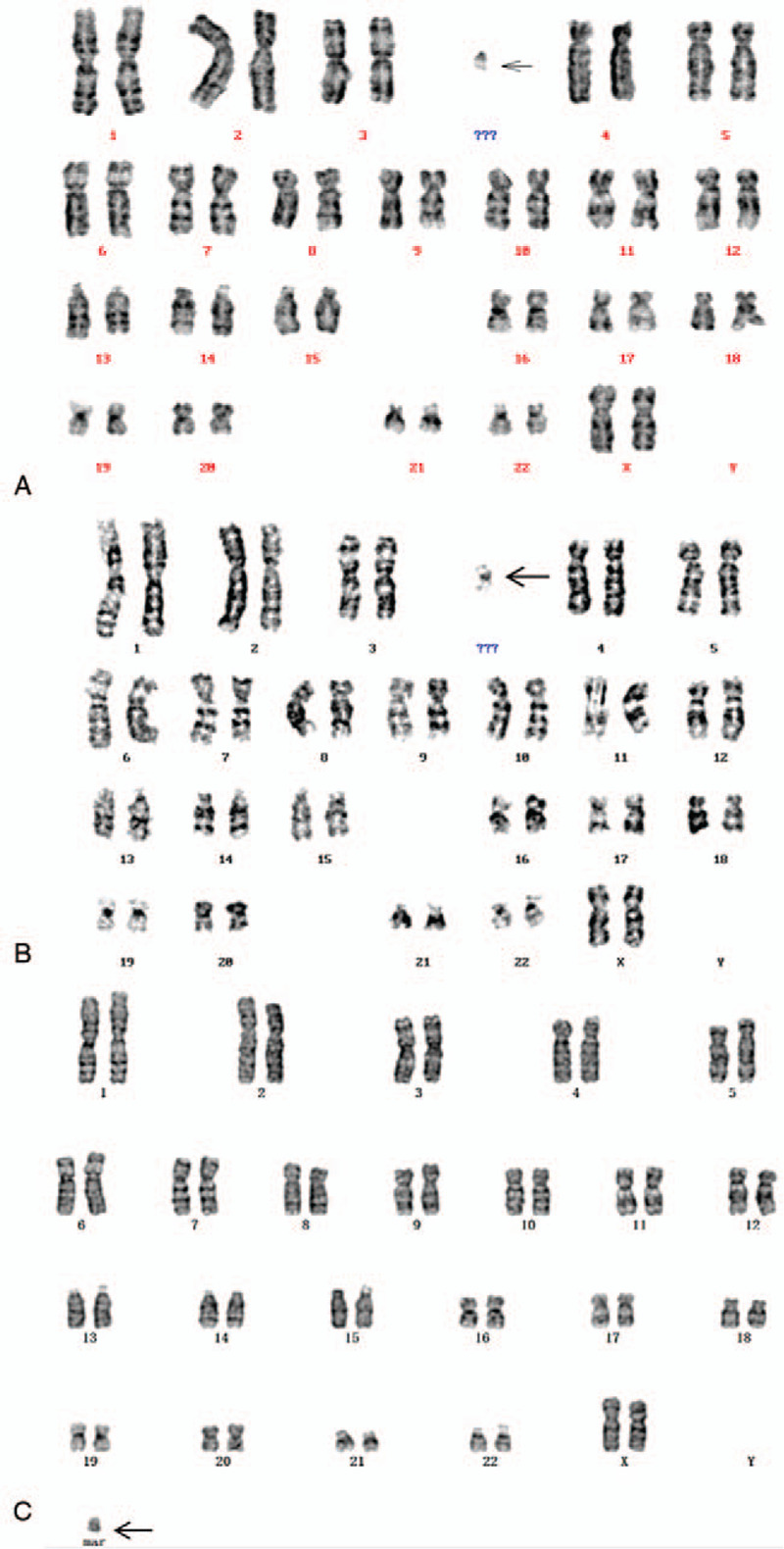
G-banding revealed the 3 patients with chromosomal karyotype 47, XX, +mar. (A) Case 1; (B) case 2; (C) case 3.

**Figure 2:**
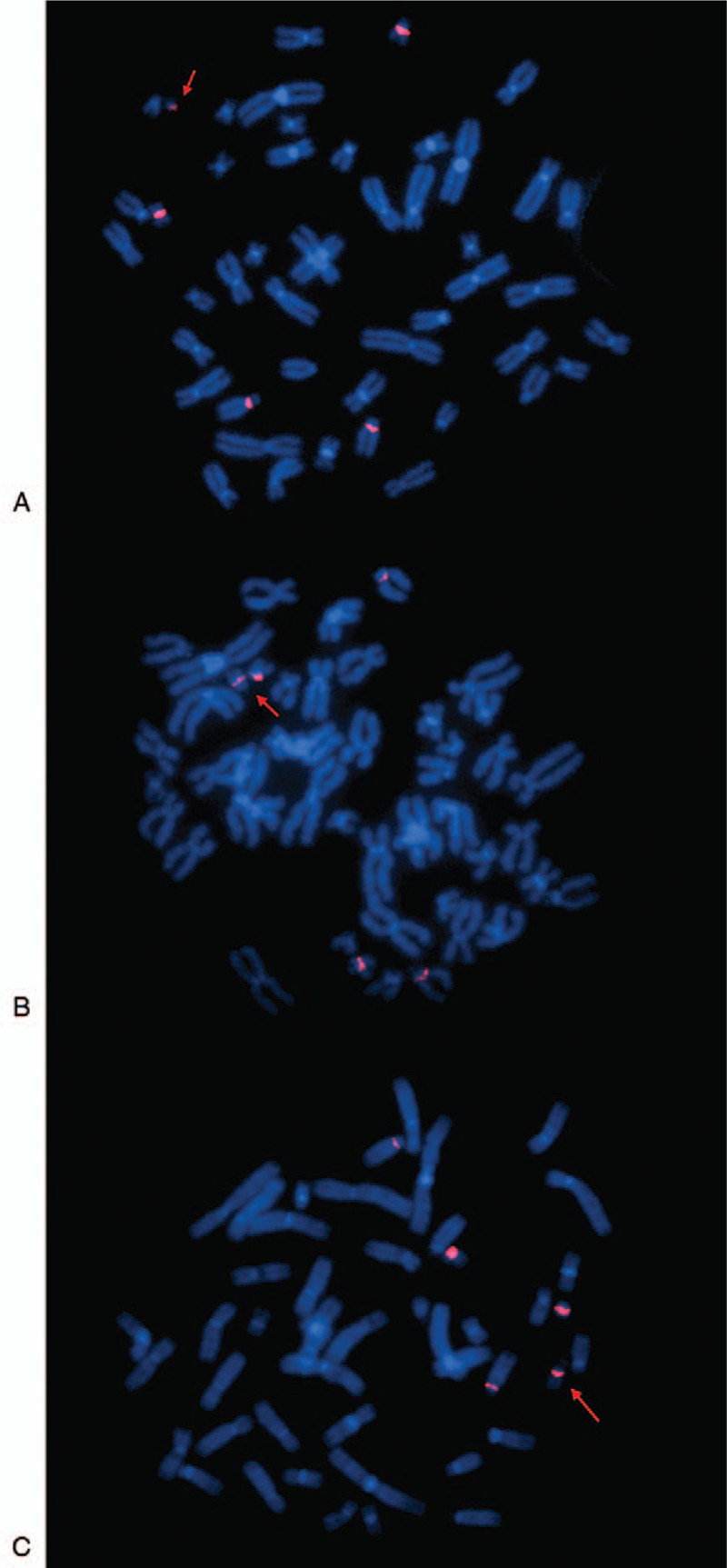
FISH results showed the sSMC were positive for D14Z1 and D22Z1 (the arrow). The marker chromosomes were finally identified as sSMC(14/22). (A) Case 1; (B) case 2; (C) case 3. FISH = fluorescence in situ hybridization; sSMC = small supernumerary marker chromosomes.

A 29-year-old, gravida 2, para 1, woman accepted in vitro fertilization (IVF) because of fertility problem and got pregnant. She and her husband were nonconsanguineous and healthy. No history of miscarriage was recorded. The woman denied being exposed to teratogenic agents, irradiation or infectious diseases, or using nicotine, alcohol or caffeine during this pregnancy. The G-banding analysis of this woman showed the karyotype of 47, XX, +mar (Fig. 1A). CMA was used to analyze the entire genetic constitutions of sSMC for all 3 patients and successfully detected a 0.49Mb gain of 5q32 region in case 1: arr[hg19] 5q32 (146,056,221–146,548,559) × 3, including the critical *PPP2R2B* gene. FISH using specific probes for chromosomes 13/21, 14/22, and 15 were applied to further identify the origination of marker chromosome. FISH analysis showed that the sSMC was positive for D14Z1/D22Z1 and negative for D13Z1/D21Z1 and D15Z1-SNRPN-PML and the results revealed that the marker chromosome was originated from sSMC(14/22) (Fig. 2A). Amniocentesis was performed at 16 weeks and chromosomal karyotype analysis showed 46, XY for the fetus. The mother continued the pregnancy and delivered a male infant at 39 weeks. The infant's birth weight was 4650 g, birth length was 58 cm and no apparent abnormalities were observed.

### 3.2 Case 2

A 24-year-old woman was referred to our center for unexplained fertility problems. She and her husband were both nonconsanguineous and healthy. No history of miscarriage was recorded. The chromosomal karyotype for this woman was 47, XX, +mar (Fig. 1B). CMA successfully detected a 0.54Mb gain of 14q32.33 region, and a 1.83Mb gain of 16p11.2 region: arr[hg19] 14q32.33 (106,328,973–106,863,507) × 3; arr[hg19] 16p11.2 (32,033,397–33,863,672) × 3, including the critical *TP53TG3D*, *TP53TG3*, *TP53TG3C*, and *TP53TG3B* genes. FISH analysis showed that the sSMC was positive for D14Z1/D22Z1 and negative for D13Z1/D21Z1 and D15Z1-SNRPN-PML and the result revealed that the marker chromosome was originated from sSMC(14/22) (Fig. 2B). The couple did not plan to choose IVF to get their offspring.

### 3.3 Case 3

A 37-year-old woman was referred to our center because of abnormal childbearing history. She and her husband were nonconsanguineous and healthy. No history of miscarriage was recorded. Her husband had the karyotype of 46, XY. The karyotype of this woman described as 47, XX, +mar (Fig. 1C). CMA successfully detected a 0.37Mb loss of 13q21.2q21.31 region and a 0.12Mb gain of Xp11.23 region: arr[hg19] 13q21.2q21.31 (62,066,753–62,439,485) × 1; arr[hg19] Xp11.23 (48,039,326–48,155,268) × 3, including the critical SSX1 and SSX5 genes. FISH analysis showed that the sSMC was positive for D14Z1/D22Z1 and negative for D13Z1/D21Z1 and D15Z1-SNRPN-PML and the result revealed that the marker chromosome was originated from sSMC (14/22) (Fig. 2C). The couple did not plan to choose IVF to get their offspring.

## 4 Discussion

Here, we reported 3 female sSMC carriers who presented various degrees of fertility problems and no apparent abnormalities. Karyotypic analysis described 47, XX, +mar for all 3 patients. CMA detected the chromosomal imbalances, showing diverse chromosomal duplications and deletions in different chromosomes. And FISH analysis finally identified the origination of sSMC for the 3 cases, which were described as sSMC(14/22).

The correlation between the sSMC and clinic phenotypes has been unclear so far.^[12]^ The phenotypic diversity of sSMC carriers was associated with the origins and genetic material. In addition, when sSMC was inherited from a parent with normal phenotypes, it was usually not harmful to the carriers’ phenotype.^[8]^ However, when sSMC was de novo, the pathogenicity was hard to predict, especially for prenatal cases.[^5^,^12^] And the risk of mental retardation and/or physical abnormalities might increased in de novo sSMC carriers.^[4]^ On the other hand, the existence of euchromatin in the sSMC often had a detrimental effect on abnormal phenotypes while the duplication of heterochromatin in sSMC were harmless.[^9^,^13^]

Currently, there were limited abnormal phenotypes associated with the existence of sSMC(14/22). To our best knowledge, additional trisomic segments of extra partial chromosome 14 may affected the patients’ clinical phenotype.^[14]^ Some abnormal clinical symptoms in sSMC(14) carriers, such as short stature, mild intellectual disability, and hypogenitalism, resulted from the duplication of 14q11.2.^[15]^ As for sSMC(22), 80% carriers were identified as non-mosaic dicentric duplications involving the euchromatic region 22q11.2, such as derivative chromosome 22 (der (22)t (11;22)(q23;q11.2)), cat-eye syndrome (inv dup (22q)).[^9^,^16^] And previous reports indicated that acrocentric sSMC was predominant in subfertile population, especially the chromosome 15, 14, and 22.^[17]^

**Figure 3:**
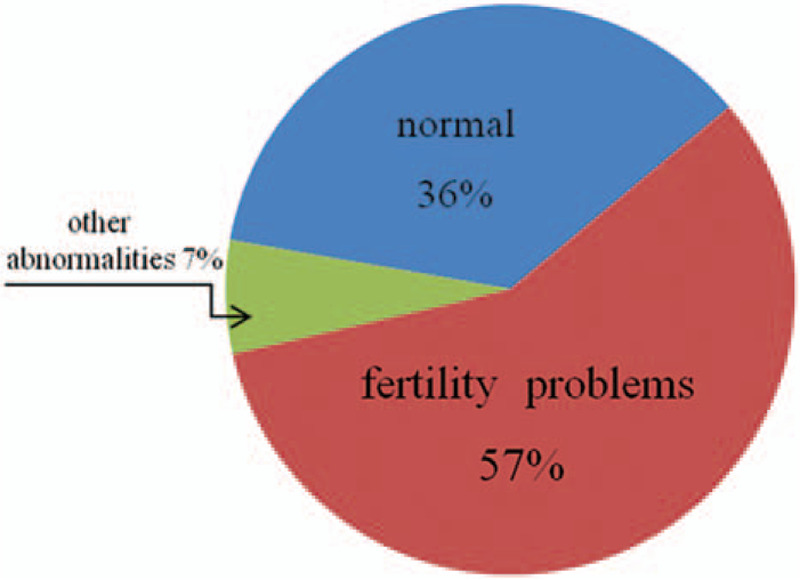
The distribution of different clinical phenotypes in female sSMC (14/22) carriers reported in the literature and present study. sSMC = small supernumerary marker chromosomes.

We summarized clinic manifestations of the adult women presenting non-mosaic sSMC (14/22) according to the review of the sSMC database, shown in Fig. 3.^[18]^ All sSMC(14/22) cases could be divided into 3 categories according to their clinical findings: fertility problems; normal phenotype; the other abnormalities. Overall, 57% (27/47) adult female had only fertility problems. 36% (17/47) carriers presented normal clinical phenotypes and only 7% (3/47) carriers presented autism, learning difficulties, or other anomaly.

To further explore the correlation between sSMC(14/22) and fertility problem, the classification and distributions of infertile adult women with non-mosaic sSMC(14/22) obtained from the sSMC database were summarized in Table 2. 67% (18/27) cases showed unexplained infertile, which made it difficult to analyze the correlation between sSMC(14/22) and fertility problems. However, it was speculated that the duplication of 14q11.1 or 22q11.1 might be associated with infertility, but more evidence was still needed. Molecular cytogenetic analysis would play a critical role in identification of sSMC and would offer better genetic counseling for sSMC carriers with infertility. Apart from the subfertility of sSMC carriers, the health of the offspring of sSMC carriers is also worth noting. It was estimated that more than half of sSMC were de novo and maternally derived sSMC might dominate the inheritance.[^3^,^13^] It was suggested that the offsprings of sSMC carriers showed sex differences: sons tend to be infertile when they inherited the sSMC from the mother, and daughters tend to be infertile when they got the sSMC from the father.^[1]^ The case 1 in our study was delivered a healthy male infant. The boy is now in a healthy state, but his future fertility ability should not be ignored and long-term follow-up should be guaranteed till his adulthood.

**Table 2:**
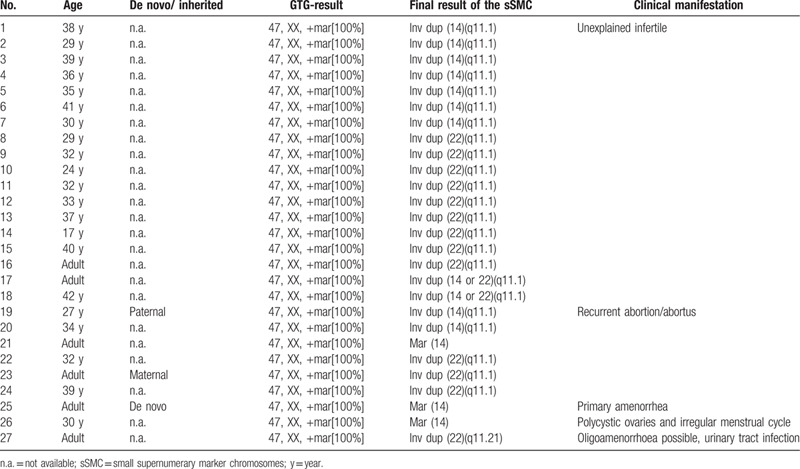
The classification and distributions of infertile adult women with non-mosaic sSMC(14/22) obtained from the sSMC database.

In addition, different chromosomal deletions and duplications were successfully detected through CMA in these cases. In case 1, CMA uncovered an additional 0.46Mb gain located at chromosome 5q32. This region contains the *PPP2R2B* gene (OMIM: 604325), the heterozygous mutation of which leads to Spinocerebellar ataxia 12, which is clinically manifested as upper limb tremor, gait ataxia, and so on.^[19]^ In case 2, no genes are involved in the region of 0.54Mb duplication at 14q32.33, and the clinical significance of this region is still unclear. The 1.83Mb gain at 16p11.2 contained *TP53TG3* (OMIM: 617482), *TP53TG3B*, *TP53TG3C*, and *TP53TG3D* genes. *TP53TG3* is one of many *TP53* target genes, which is a transcription factor involving in cell cycle arrest, apoptosis, DNA repair, chromosomal stability, and inhibition of angiogenesis.^[20]^ In case 3, the deleted 13q21.2q21.31 region is not found to contain genes. *SSX1* (OMIM:312820) and *SSX5* (OMIM:300327) genes, located in Xp11.23, were members of *SSX* gene family expressed in testis.^[21]^ Generally speaking, there were no evidence to support the fact that these chromosomal imbalances have potential association with female infertility and they were inclined to be likely benign variants.

For female sSMC carriers with fertility problems, assisted reproductive techniques (ART) are effective approaches to get their offsprings. Moreover, the application of preimplantation genetic diagnosis (PGD) can detect marker chromosomes in embryos using the special probes, which would be useful in distinguishing the normal and balanced embryos for further embryo transfer.^[22]^

## 5 Conclusion

In summary, we presented 3 adult female carrying marker chromosomes through G-banding, CMA, and FISH analysis. And they were finally identified as sSMC(14/22). All 3 carriers had normal phenotypes except for fertility problems, which were rarely reported in clinic. For sSMC carriers with fertility problems, tradition cytogenetics and molecular cytogenetic techniques could be considered to be applied simultaneously for more detailed characterization of these marker chromosomes, which would offer comprehensive fertility assessment and guidance.

## Author contributions

**Data curation:** Xiaonan Hu.

**Methodology:** Qi Xi, Leilei Li.

**Project administration:** Ruizhi Liu.

**Supervision:** Ruizhi Liu.

**Validation:** Ruizhi Liu.

**Writing – original draft:** Meiling Sun.

**Writing – review & editing:** Han Zhang, Hongguo Zhang.
